# Treatment and survival of early non-metastatic breast cancer in men: real world data from a population-based registry

**DOI:** 10.1007/s00404-025-08139-8

**Published:** 2025-08-18

**Authors:** D. Jakob, D. Dannehl, H. Endres, L. Jansen, S. Hermann, A. D. Hartkopf, S. Huwer, L. Jung, O. Thijssen, I. Juhasz-Böss, F. A. Taran

**Affiliations:** 1 Department of Obstetrics and Gynecology, Faculty of Medicine, University Medical Center Freiburg, University of Freiburg, Hugstetterstr. 55, 79106 Freiburg im Breisgau, Germany; 2 Department of Women’s Health, Tuebingen University Hospital, Tübingen, Germany; 3 German Cancer Research Center (DKFZ), Epidemiological Cancer Registry Baden-Württemberg, Heidelberg, Germany; 4 Department of Gynecology and Gynecologic Oncology, Center for Integrated Oncology Aachen Bonn Cologne Düsseldorf, Medical Faculty and University Clinic of Cologne, University of Cologne, Cologne, Germany

**Keywords:** Male breast cancer, Real-world data, Surgery

## Abstract

**Aim:**

This study aimed to characterize a cohort of male patients with non-metastatic breast cancer, specifically focusing on tumor characteristics, treatment strategies, and determinants of overall survival.

**Methods:**

Data for this study were obtained from the Baden-Württemberg Cancer Registry, encompassing male patients diagnosed with breast cancer between 2015 and 2023. A total of 470 patient records were included. We described patient and tumor characteristics using descriptive statistics. Overall survival was analyzed using Kaplan–Meier survival curves and Cox proportional hazards regression models to identify significant determinants.

**Results:**

In our cohort of male patients with non-metastatic breast cancer, luminal subtype was the predominant tumor biology, accounting for 90% of cases. HER2-positive tumors were observed in 9% of patients, while triple-negative tumors were rare, with only four cases identified. Regarding tumor staging, 81.7% of patients were diagnosed at T1 or T2 stages. However, a substantial proportion (48.7%) presented with clinically involved lymph nodes, and 27.1% were diagnosed at UICC stage III. The five-year overall survival rate for the cohort was 73.7%. Treatment analysis revealed that 86% of patients underwent surgical intervention. Mastectomy combined with sentinel lymph node dissection was the most frequent surgical procedure, performed in 50.6% of cases. Adjuvant radiotherapy was administered to 72.8% of patients. Cox regression analysis identified age, nodal status, and surgical intervention as significant determinants of overall survival.

**Supplementary Information:**

The online version contains supplementary material available at 10.1007/s00404-025-08139-8.

## What does this study add to the clinical work

**Table:** 

This study provides contemporary, real-world evidence on tumor biology, treatment patterns, and survival outcomes in men with non-metastatic breast cancer, a rare and understudied population. It highlights that tumor size, nodal status, and surgical treatment remain key prognostic factors, reinforcing their importance in clinical decision-making.	

## Introduction

Breast cancer in men is a rare disease with approximately 700 new cases diagnosed every year in Germany and an increasing incidence over the last years [1]. One possible reason could be obesity, which can lead to a higher estrogen synthesis as a contributing risk factor [2]. Due to the lack of screening and less knowledge about breast cancer among men, the disease is more often diagnosed at the more advanced Union Contre le Cancer (UICC) stages III and IV [3]. The five-year overall survival rate after breast cancer is lower (77 percent) for men compared to women (88 percent) [1, 4].

Genetic predisposition, especially BRCA1 and BRCA2, is more common in men with breast cancer [5]. Biologically, more than 90% of men with breast cancer express the estrogen receptor (ER). Literature on the human epidermal growth factor receptor 2 (HER2)-status is variable, with HER2-overexpression being present in 0–37% of cases [6–9]. ER-positive HER2-negative tumors are classified as luminal tumors.

Due to the rarity of the disease, there is very little evidence from randomized studies concerning diagnostic, biological characteristics, and treatment of breast cancer in men. Treatment recommendations rely on data from the treatment of postmenopausal women with breast cancer [10], despite the fact that there are biological differences in male breast cancer compared with female breast cancer [8, 11].

Surgical treatment of non-metastatic breast cancer in men consists mainly of mastectomy and axillary lymph node dissection, in some cases complemented with radiation therapy [12, 13]. If lymph nodes are affected, adjuvant chemotherapy is recommended, and in case of HER2-overexpression, anti-HER2-therapy is recommended [14]. The standard endocrine therapy in men is Tamoxifen; aromatase inhibitors lead to a higher mortality in men [15]. However, Tamoxifen is also associated with side effects in men, such as sexual dysfunction, and there is a high therapy drop-out rate [16]. There is also limited data on newer therapy options for high-risk non-metastatic breast cancer, for example, the use of cyclin-dependent kinase 4/6 inhibitors or Olaparib in patients with BRCA1 or BRCA2 mutations, even though men are more likely to meet the criteria for extended treatment with these substances [17].

To improve knowledge, there have been initiatives to develop registries to collect data on treatment and survival of breast cancer in men [18]. A possibility to analyze data on cancer treatment in Germany is to use cancer registries. In Baden-Württemberg, the third largest federal state in Germany with a population of approximately 11 million, there is a legal obligation to report diagnosis, treatment, and progression of neoplasms since 2011 [19].

The objective of this study is to analyze population-based data on the treatment and survival of men with breast cancer in Baden-Württemberg using the statewide cancer registry.

## Methods

We retrospectively selected and analyzed population-based cancer registry data in collaboration with the Baden-Württemberg Cancer Registry. Data from men diagnosed with breast cancer between January 1, 2015, and December 31, 2022, were included in this analysis.

We did not have access to individual patient data because the data had already been compiled by the Baden-Württemberg Cancer Registry. The data are anonymized by the registry and any information that could be used to identify individual patients or hospitals had already been removed. Therefore, neither ethical approval nor informed patient consent was required. Due to the retrospective nature of our analysis, certain tumor characteristics were not available to us as they were not documented, including genomic risk profiling, Ki-67 proliferation index, and the BRCA1 and BRCA2 mutation status.

**Fig. 1:**
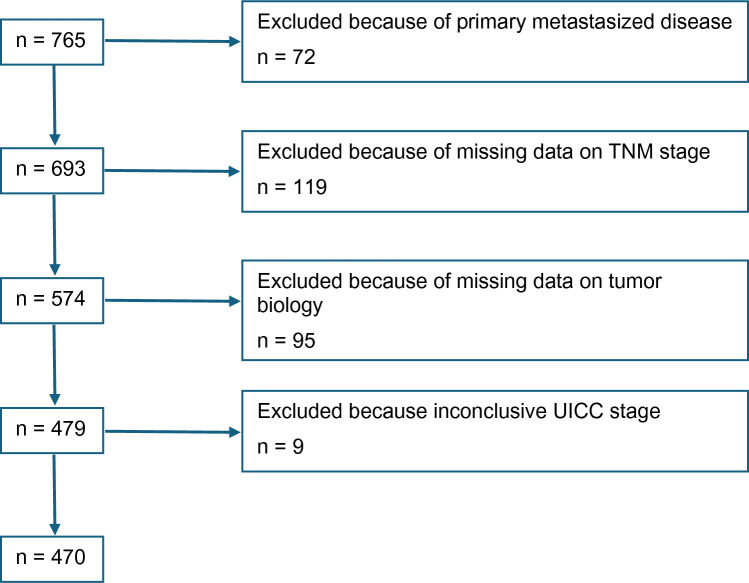
Consort diagram: of 765 screened patient cases 295 were excluded due to primary metastasized disease, missing data, or inconclusive UICC stage. Data from 470 patients were analyzed

Data from 765 men with breast cancer were considered for this analysis. Exclusion criteria were distant metastatic disease, carcinoma in situ, and missing or inconclusive data. Tumors were considered luminal if expression of the estrogen and/or progesterone receptor had been reported as positive and there was no HER2 overexpression. Data from 470 men with breast cancer were finally analyzed (Fig. 1, consort diagram).

In a subset of patients, the exact type of surgical procedure was not recorded, although a pathological TNM stage was available. Consequently, these individuals were grouped together with patients for whom a specific procedure had been coded. The combined group was then compared to patients who did not undergo any surgery. In this analysis, the TNM stage was presented in a summarized form. Whenever possible, the pathological TNM stage was reported. If the pathological TNM stage was not available, the clinical TNM stage was used instead.

Patients’ vital status was last assessed end of 2022, based on linkage with residents’ registration office data. Survival data was evaluated using Kaplan–Meier statistics and log-rank tests. To account for immortal time bias, a survival analysis was conducted for all patients, as well as for those who were alive one year after diagnosis. Factors influencing overall survival were evaluated using a Cox regression analysis. For statistical comparisons, the significance level was set to α = 0.05.

Data processing and statistical analyses were performed using Jupyter Notebook (Version 6.3.0, Project Jupyter, open-access and community developed) on Anaconda (Version 3.0, Anaconda Inc., Austin, TX, USA), with the Python extension packages pandas (Version 1.4.1, open-access and community developed) and numeric Python (Version 1.22.2, open-access and community developed).

## Results

In our patient cohort, the luminal subtype predominated, accounting for 90.0% of tumors (423/470; see Table 1). Due to their low numbers, triple-negative tumors (*n* = 4) were excluded from further analysis. Notably, no cases of HER2-positive/hormone receptor-negative (HR-/HER2 +) tumors were identified. 43/470 patients had an HR + /HER2- tumor biology (9.2%). A key distinction between luminal and HER2-positive tumors was their histopathological grade at diagnosis. Only 26.7% of luminal tumors were poorly differentiated (Grade 3), compared to a significantly higher 48.8% in the HER2-positive group. Other fundamental tumor characteristics showed no significant differences between these two groups. Overall, the majority of tumors were diagnosed at T1 or T2 stage (81.7% of all cases), and roughly half of the patients presented with no lymph node involvement (51.3%).

**Table 1:** Cohort tumor baseline characteristics: age, biology, grading, primary tumor size, nodal status and UICC stage of all patients, luminal and HER2 + patients

Cohort tumor baseline characteristics			
	*n* = 470	Luminal	HER2 +
Tumor biology			
Luminal	423 (90.0%)		
HER2 +	43 (9.15%)		
Triple neg	4 (0.85%)		
Mean age (years ± SD)	69.48 ± 12.06	69.75 ± 11.81	66.77 ± 13.66
Grading	*n* = 463	*n* = 416	*n* = 43
G1	34 (7.3%)	32 (7.7%)	2 (4.7%)
G2	295 (63.7%)	273 (65.6%)	20 (46.5%)
G3	134 (29.0%)	111 (26.7%)	21 (48.8%)
Primary tumor size	*n* = 470	*n* = 423	*n* = 43
T1	183 (38.9%)	167 (39.5%)	14 (32.6%)
T2	201 (42.8%)	179 (42.3%)	20 (46.5%)
T3	12 (2.6%)	10 (2.4%)	2 (4.7%)
T4	74 (15.7%)	67 (15.8%)	7 (16.3%)
Nodal status	*n* = 467	*n* = 421	*n* = 42
N0	240 (51.3%)	215 (51.1%)	22 (52.3%)
N1	161 (34.4%)	149 (35.4%)	12 (28.6%)
N2	52 (11.1%)	46 (10.9%)	5 (11.9%)
N3	14 (3.0%)	11 (2.6%)	3 (7.1%)
UICC stage	*n* = 470	*n* = 423	*n* = 43
IA	125 (26.6%)	113 (26.7%)	11 (25.6%)
IB	7 (1.5%)	6 (1.4%)	1 (2.3%)
IIA	135 (28.7%)	122 (28.8%)	11 (25.6%)
IIB	75 (16.0%)	69 (16.3%)	6 (14.0%)
IIIA	43 (9.1%)	37 (8.7%)	5 (11.6%)
IIIB	71 (15.1%)	65 (15.4%)	6 (14.0%)
IIIC	14 (3.0%)	11 (2.6%)	3 (7.0%)

*SD* standard deviation

The majority of patients (85.2%) received a modified radical mastectomy. Lumpectomy was rare in only 4.5% of cases. Most patients received axillary surgery, with sentinel lymph node biopsy being the most frequent (68.9%), followed by axillary lymph node dissection (26.2%). In our cohort, only 4.9% of patients did not undergo axillary surgery; however, this proportion was higher in the HER2 + subgroup at 13.5%. Additionally, 5.4% of patients did not receive any surgical treatment, with a slightly higher proportion of 7.5% in the HER2 + group. Overall, 72.8% of patients received radiation therapy (See Table 2: Cohort treatment data).

**Table 2:** Treatment data (operative procedure, lymph node procedure and radiation of all patients, luminal and HER2 + patients

Cohort treatment data			
Type of breast surgery	All, *n* = 470	Luminal, *n* = 423	HER2 + , *n* = 43
MRM	381 (85.2%)	343 (89.6%)	34 (85.0%)
Lumpectomy	20 (4.5%)	17 (4.4%)	3 (7.5%)
No breast surgery	43 (9.6%)	23 (6.0%)	3 (7.5%)
Not documented	23	40	3
Lymph node procedure			
SLN	255 (68.9%)	230 (69.9%)	22 (59.5%)
ALND	97 (26.2%)	86 (26.1%)	10 (27.0%)
No lymph node procedure	18 (4.9%)	13 (4.0%)	5 (13.5%)
Not documented	100	94	6
Operative procedure			
MRM + SLN	238 (55.7%)	215 (56.1%)	20 (54.1%)
MRM + ALND	93 (21.8%)	83 (21.7%)	9 (24.3%)
Only MRM	50 (11.7%)	45 (11.7%)	5 (13.5%)
Lumpectomy + SLN	15 (3.5%)	13 (4.0%)	2 (5.4%)
Lumpectomy + ALND	3 (0.7%)	2 (0.5%)	1 (2.7%)
Lumpectomy only	2 (0.5%)	2 (0,5%)	0
Only Sentinel	2 (0.5%)	2 (0.5%)	0
Only ALND	1 (0.2%)	1 (0.3%)	0
No operative treatment	23 (5.4%)	20 (5.2%)	3 (7.5%)
Not documented	43	40	3
Radiation			
Yes	342 (72.8%)	310 (73.3%)	30 (69.8%)
No	128 (27.2%)	113 (26.7%)	13 (30.2%)

Percentages were calculated based on the total patient number subtracted with the number of not documented cases

*MRM* modified radical mastectomy, *SLN* sentinel lymphonodectomy, *ALND* axillary node dissection

**Fig. 2:**
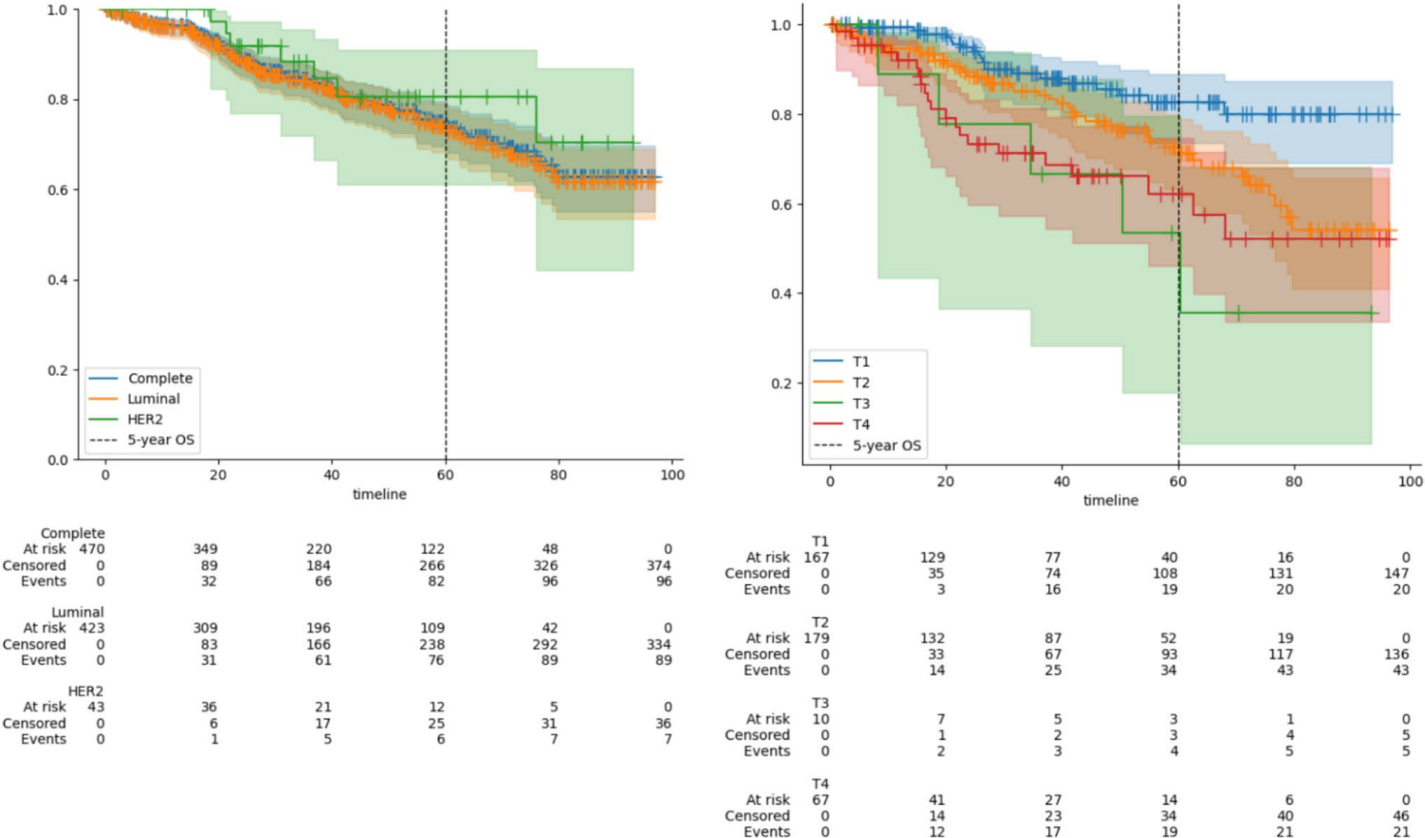
**A** Overall survival of the total cohort and stratified after tumor biology. **B** Overall survival of the cohort with luminal subtype

The survival data of the cohort were evaluated based on tumor biology, tumor size, nodal status, and received therapy. The five-year overall survival rate in this cohort was 75.0%. Specifically, the five-year overall survival for patients with luminal tumors was 74.1%, while in the smaller subgroup of HER2-positive tumors, it was 80.6%. The analysis of survival in luminal tumors according to tumor size revealed a significantly higher survival rate in patients with T1-stage tumors (five-year overall survival of 82.7%) compared to those with T3 (53.3%) and T4 (62.2%) stages (Fig. 2).

**Fig. 3:**
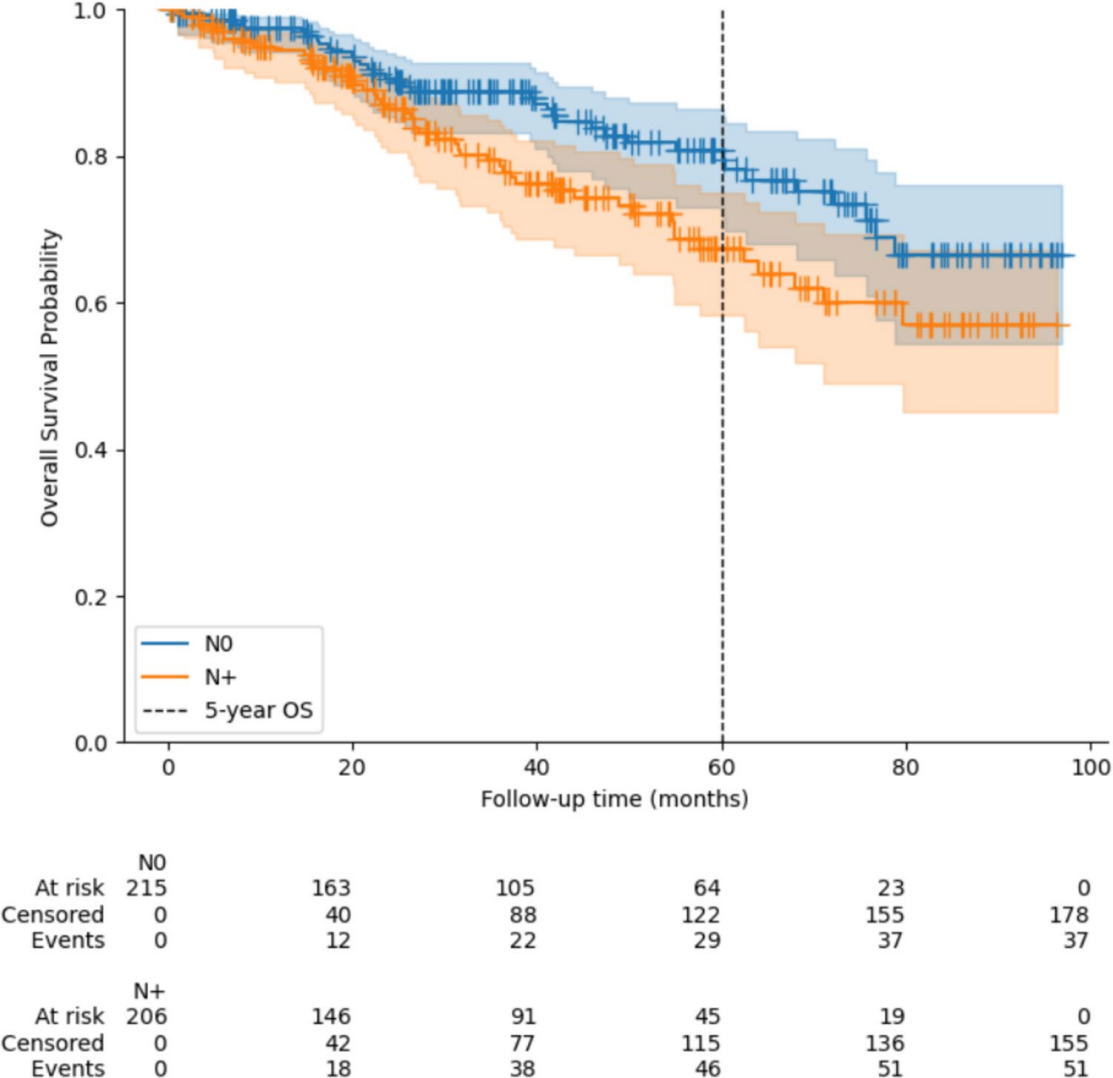
Overall survival of the cohort with luminal subtype stratified after nodal status

A comparison between patients with luminal tumors and positive nodal status and those without affected lymph nodes demonstrated that patients without affected lymph nodes had a five-year overall survival rate of 80.7%, whereas those with positive lymph nodes had a survival rate of 67.4%. This difference was statistically significant (*p* = 0.02) (Fig. 3).

**Fig. 4:**
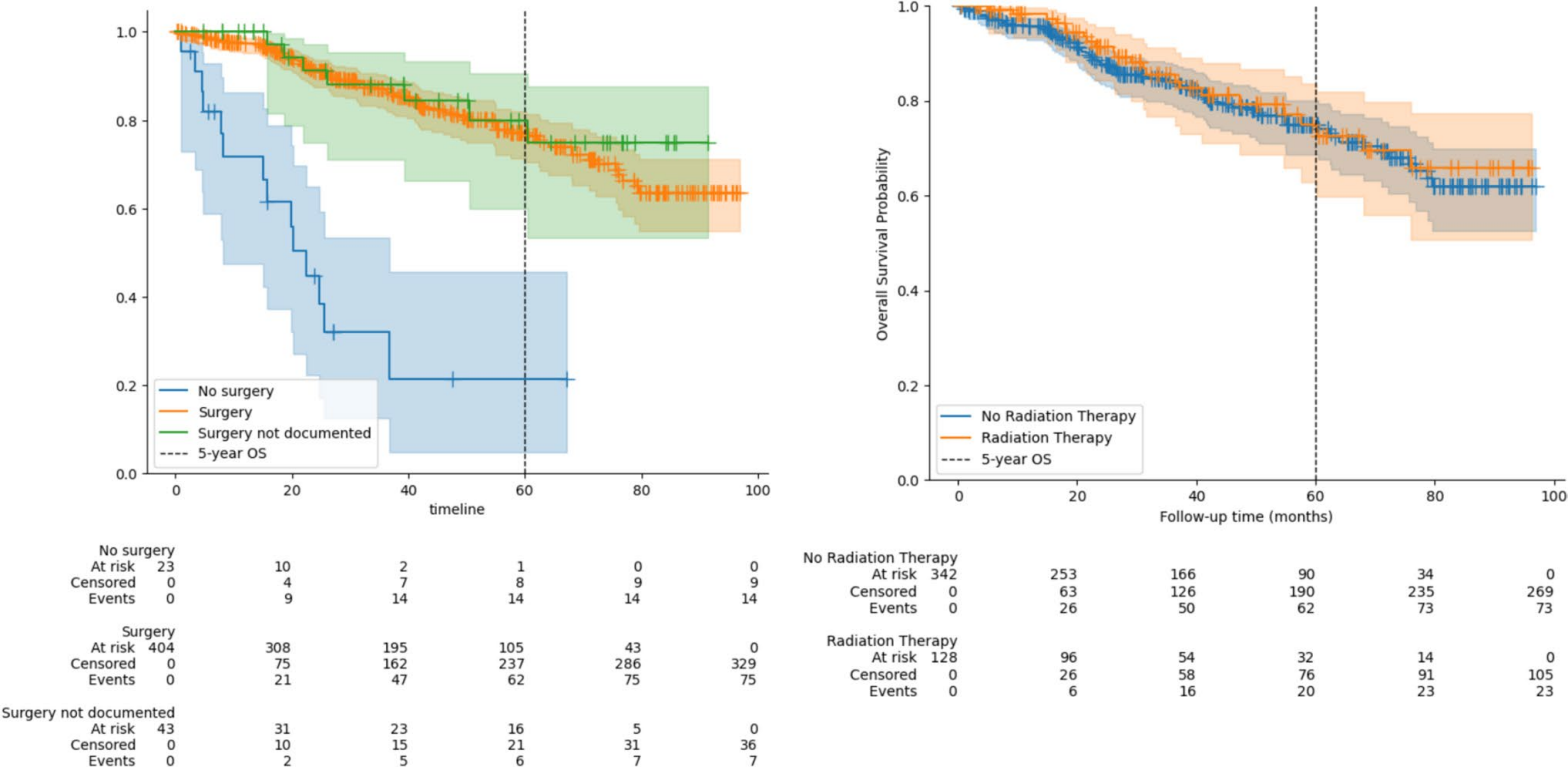
Kaplan–Meier curves of survival of patients with breast cancer stratified by **A** surgical treatment received and **B** radiation therapy received

Patients who underwent surgical treatment demonstrated a survival benefit (Fig. 4A). The five-year overall survival rate among patients who received any form of surgical treatment was 77.1% and 79.9% (surgery and surgery not documented), compared to 21.3% in those for whom it was documented that surgery was not performed. This difference was statistically significant (*p* < 0.005). The 43 patients with missing documentation regarding surgical treatment exhibited a survival rate comparable to those who underwent surgery (*p* = 0.52). In contrast, no significant differences were observed when comparing survival rates between patients stratified by whether they had received radiotherapy (74.9%) or not (74.9%) (*p* = 0.6) (Fig. 4B). We also separately analyzed the impact of surgical treatment and radiotherapy for the 349 cases where there was at least 1 year follow-up documented (see supplementary figures).

A Cox regression analysis was conducted to evaluate the impact of various factors on the dependent variable, overall survival. The independent variables included age, tumor grade, T-stage, N-stage, adjuvant radiotherapy, surgical treatment, and chemotherapy. The analysis identified three significant prognostic factors. Age was negatively associated with survival, meaning that increasing age was linked to a higher risk of mortality (hazard ratio: 1.06, 95% confidence interval (CI): 1.03–1.09). Similarly, N-stage had a negative impact, with higher nodal involvement correlating with an increased risk of mortality (hazard ratio: 1.94, 95% CI: 1.47–2.55). In contrast, surgical treatment was found to be a protective factor, significantly reducing the risk of mortality (hazard ratio: 0.15, 95% CI: 0.08–0.28); see Table 3.

**Table 3:** Cox regression analysis of the impact of age, tumor grade, T-stage, N-stage, radiotherapy, surgical treatment and chemotherapy on survival

Variable	Hazard ratio	95% confidence interval	*P*-value
Age (Per year)	**1.06**	**1.03–1.09**	** < 0.0001**
Grading	1.33	0.92–1.94	0.13
T-stage	1.14	0.93–1.40	0.19
N-stage	**1.94**	**1.47–2.55**	** < 0.0001**
Radiation therapy (Y/N)	0.71	0.43–1.16	0.18
Surgery (Y/N)	**0.15**	**0.08–0.28**	** < 0.0001**
Chemotherapy (Y/N)	0.63	0.35–1.13	0.12

Bold type indicates statistical significance

## Discussion

This study investigated real-world data on the treatment and survival of 470 men with non-metastatic breast cancer in Baden-Württemberg using the statewide cancer registry data. The main findings of the study are that tumor stage, determined by tumor size and nodal status, as well as surgical treatment, are significant indicators of survival in this patient cohort.

The five-year overall survival in our study of men with non-metastatic breast cancer is 75.0%. Breast cancer survival data for men in Germany, including patients with metastasized disease, show a five-year overall survival of 62%, whereas the five-year overall survival of women is higher (79%) despite including patients with metastatic breast cancer [20]. Our analysis can confirm that a high percentage of men with breast cancer are diagnosed at a later stage compared to women with breast cancer. In our study, 27.1% of men were diagnosed with UICC stage III breast cancer, compared to only 11% of women when restricting to non-metastatic disease [20]. In our study, this is also associated with worse outcomes in accordance with previously published data [11, 12].

Our data on the tumor biology of male breast cancer aligns with previously published data (90.0% luminal tumors, 9.15% HER2 + tumors, 0.85% triple-negative tumors) [12]. However, due to a lack of documentation within the registry, the present study did not provide any information on BRCA-mutation status. Nevertheless, a higher rate of BRCA2 mutations in men compared to women may be associated with more aggressive cancers and thus a reduced survival rate [21, 22].

The present analysis confirms the impact of nodal status on overall survival in men with non-metastatic breast cancer, whereas tumor size did not have an independent impact on survival in the Cox regression analysis. In our dataset, 34.4% of patients were diagnosed with clinically positive lymph nodes, but only 26.2% underwent axillary dissection. Our data cannot answer the question of whether this discrepancy is due to missing information or to individual treatment concepts. The potential de-escalation of lymph node procedures is discussed in breast cancer with the possibility of a targeted axillary dissection in the setting of clinical remission after neoadjuvant therapy [23]. Omission of axillary staging in patients with small tumors (T1 and T2) and no clinically suspicious lymph nodes has also been shown to benefit patients in terms of side effects such as lymphedema or shoulder pain [24]. The safety of this de-escalation of treatment should also be evaluated for male breast cancer patients.

Another result of this study is the association of surgery with longer survival after breast cancer. Approximately 5% of patients in our study did not undergo surgery, which is highly comparable to an analysis from the United States [25]. Reasons for not undergoing surgery could be poor general health, increased age, or fear of surgical complications and long-term side effects such as lymphedema [26]. Supplementary Table 1 shows the baseline patient characteristics of patients without breast surgery. It could show that these patients are significantly older (*p* < 0.0001) compared to patients that underwent surgery. Unfortunately, due to a limitation of the dataset, the patients that did not undergo surgery could not be further characterized regarding comorbidities or patients’ preferences. However, our data indicate that the omission of surgery is associated with a worse five-year overall survival while radiotherapy did not show any association with the five-year overall survival.

Our study does not provide information on the use of targeted therapies in the adjuvant setting for breast cancer in men. New oral anticancer agents have shown to increase survival in women with breast cancer, but only few men were included in the studies [27–29]. Our data are consistent with previously published data that more men than women may be eligible for post-neoadjuvant targeted therapy due to the higher stage at diagnosis, and that the effects of this therapy on the prognosis of men with breast cancer should be analyzed [17].

By nature, this retrospective analysis of real-world data has its limitations. There are missing data and in some cases data were inconsistent. There were patients for whom no surgery was coded, but who still had a pT-stage documented in the registry. An exploratory analysis could demonstrate that these patients have a similar survival pattern compared to patients that underwent surgery. Hence, these two patient cohorts were grouped and analyzed together. This warrants caution in drawing definitive conclusions from these data. A strength of this study is the large collective (470 cases over 8 years), given the rarity of the disease, with detailed clinical information on tumor biology, subtype, treatment, and outcome.

Male patients are underrepresented in classical clinical studies; thus, it is important to characterize this marginalized patient group using real-world studies. Since data from prospective clinical studies are rare, it is desirable to include these patients in observational studies to collect more reliable real-world data in prospective clinical registries. More detailed data on adjuvant treatment, endocrine therapy, quality of life, and patient compliance should be collected.

## Conclusion

Real-world data on the treatment and survival of 470 men with non-metastatic breast cancer demonstrates that higher age, positive nodal status, and omission of surgical treatment are associated with a reduced five-year overall survival.

## Supplementary Information

Below is the link to the electronic supplementary material.

Supplementary file1 (DOCX 159 KB)

## Data Availability

Data for this analysis was provided by the Baden-Wuerttemberg cancer registry.
