# Supplementary material for: Treatment and survival of early non-metastatic breast cancer in men: real world data from a population-based registry
Source: Arch Gynecol Obstet. 2025 Aug 18;312(5):1561–9. doi: 10.1007/s00404-025-08139-8 (PMC12589361; doi:10.1007/s00404-025-08139-8)
Supplement: Supplementary file 1 — Supplementary file1 (DOCX 159 KB) [file 404_2025_8139_MOESM1_ESM.docx]

### Supplementary figures


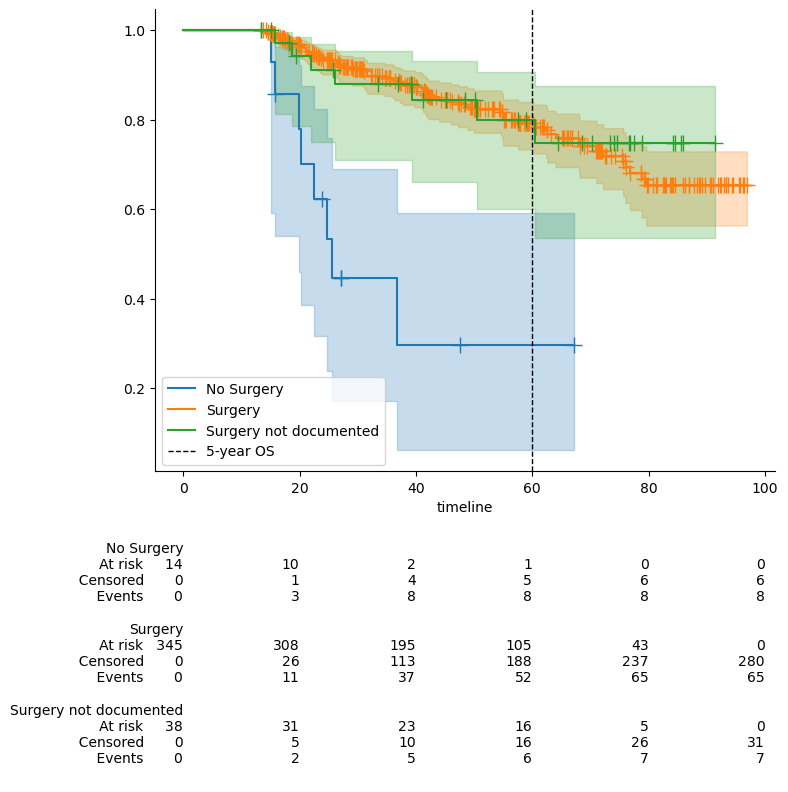


Supplementary figure 1: Kaplan-Meier curves of survival of patients with breast cancer stratified by surgical treatment, only cases with at least 1 year follow-up


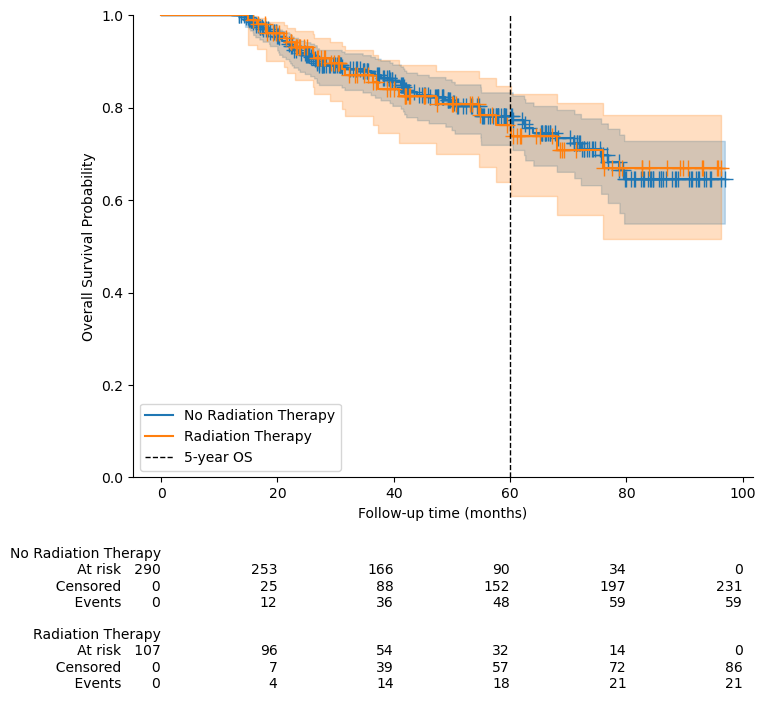


Supplementary figure 2: Kaplan-Meier curves of survival of patients with breast cancer stratified by radiation therapy received, only cases with at least 1 year follow-up

| **Tumor baseline characteristics – no surgery only** | | |
| --- | --- | --- |
|  | N=23 |  |
| Tumor biology   - Luminal - HER2+ - Triple-neg | 20 (87.0%)  3 (13.0%)  0 |  |
| Age | 80.65 ±9.83 years |  |
| Grading   - G1 - G2 - G3 - Unknown | 1 (4.3%)  16 (69.6%)  5 (21.7 %)  1 (4.3%) |  |
| Primary tumor size   - T1 - T2 - T3 - T4 | 6 (26.1%)  10 (43.5%)  1 (4.3%)  6 (26.1%) |  |
| Nodal status   - N0 - N1 - N2 - N3 | 12 (52.2%)  10 (43.5%)  1 (4.3%)  0 (0%) |  |
| UICC stage   - IA - IB - IIA - IIB - IIIA - IIIB - IIIC | 5 (21.7%)  0 (0%)  7 (30.4%)  4 (17.4 %)  1 (4.3%)  6 (26.1%)  0 (0 %) |  |

Supplementary Table 3: Tumor baseline characteristics – no surgery only: age, biology, grading, primary tumor size, nodal status and UICC stage of all patients, luminal and HER2+ patients.
